# Robust In Vitro and In Vivo Immunosuppressive and Anti-inflammatory Properties of Inducible Caspase-9-mediated Apoptotic Mesenchymal Stromal/Stem Cell

**DOI:** 10.1093/stcltm/szab007

**Published:** 2022-03-03

**Authors:** Paola Alejandra Romecín, Meritxell Vinyoles, Belén López-Millán, Rafael Diaz de la Guardia, Noemi M Atucha, Sergi Querol, Clara Bueno, Raquel Benitez, Elena Gonzalez-Rey, Mario Delgado, Pablo Menéndez

**Affiliations:** 1 Josep Carreras Leukemia Research Institute, Barcelona, Spain; 2 RICORS-TERAV, ISCIII, Madrid, Spain; 3 GENYO, Centro Pfizer-Universidad de Granada-Junta de Andalucía de Genómica e Investigación Oncológica, Granada, Spain; 4 Departamento de Fisiologia Humana, Facultad de Medicina, Murcia, Spain; 5 Banc de Sang i Teixits, Barcelona, Spain; 6 CIBERONC, ISCIII, Barcelona, Spain; 7 Instituto de Parasitologia y Biomedicina López-Neyra (IPBLN-CSIC), Armilla, Granada, Spain; 8 Institució Catalana de Recerca i Estudis Avançats (ICREA), Barcelona, Spain

**Keywords:** BM-MSC, WJ-MSC, iCasp9 switch, immunosuppression, anti-inflammatory, colitis in vivo model

## Abstract

Mesenchymal stromal stem/cells (MSC) therapies are clinically used in a wide range of disorders based on their robust HLA-independent immunosuppressive and anti-inflammatory properties. However, the mechanisms underlying MSC therapeutic activity remain elusive as demonstrated by the unpredictable therapeutic efficacy of MSC infusions reported in multiple clinical trials. A seminal recent study showed that infused MSCs are actively induced to undergo apoptosis by recipient cytotoxic T cells, a mechanism that triggers in vivo recipient-induced immunomodulation by such apoptotic MSCs, and the need for such recipient cytotoxic cell activity could be replaced by the administration of ex vivo-generated apoptotic MSCs. Moreover, the use of MSC-derived extracellular vesicles (MSC-EVs) is being actively explored as a cell-free therapeutic alternative over the parental MSCs. We hypothesized that the introduction of a “suicide gene” switch into MSCs may offer on-demand in vivo apoptosis of transplanted MSCs. Here, we prompted to investigate the utility of the iCasp9/AP1903 suicide gene system in inducing apoptosis of MSCs. iCasp9/AP1903-induced apoptotic MSCs (MSC^iCasp9+^) were tested in vitro and in in vivo models of acute colitis. Our data show a very similar and robust immunosuppressive and anti-inflammatory properties of both “parental” alive MSC^GFP+^ cells and apoptotic MSC^iCasp9+^ cells in vitro and in vivo regardless of whether apoptosis was induced in vivo or in vitro before administering MSC^iCasp9+^ lysates. This development of an efficient iCasp9 switch may potentiate the safety of MSC-based therapies in the case of an adverse event and, will also circumvent current logistic technical limitations and biological uncertainties associated to MSC-EVs.

## Graphical Abstract

**Graphical Abstract:**
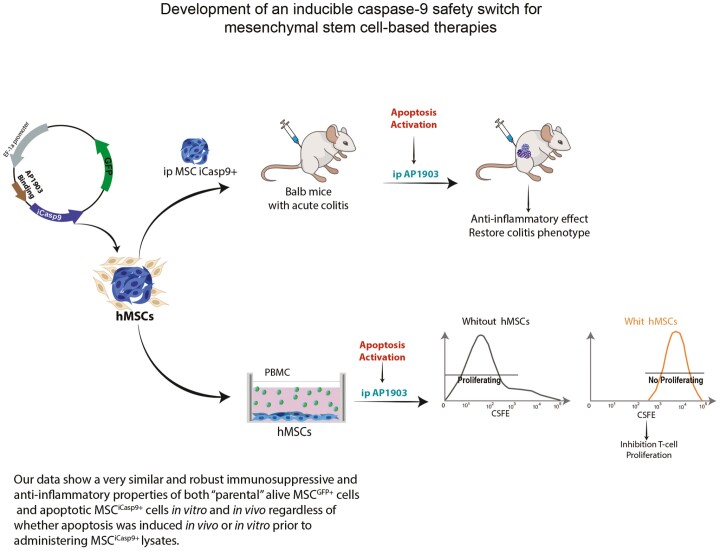

## Introduction

Mesenchymal stromal stem/cells (MSC) are multipotent mesodermal progenitor cells that can differentiate into many lineages of mesenchymal tissues.^1,2^ They can be isolated from a variety of tissues including bone marrow (BM), Wharton Jelly (WJ), cord blood, amniotic fluid and amniotic membrane, skeletal muscle, adipose tissue, dental pulp, and placenta among others.^2,3^ Beyond their multi-lineage differentiation capacity, MSCs lack immunogenicity, and display robust immunosuppressive and anti-inflammatory properties, a set of features that have prompted their wide use in cell therapies for a range of autoimmune and inflammatory diseases, graft failure, and graft-versus-host-disease (GvHD) in the context of hematopoietic stem cell transplantation, osteoarticular disorders, heart and pulmonary fibrotic disorders and even SARS-Cov-2.^4,5^ Although the therapeutic efficacy of MSCs has been demonstrated in preclinical disease models and in multiple human phase I/II clinical trials, progress beyond phase III trials has not been achieved.^6^

The mechanisms underlying MSC therapeutic activity remain elusive.^6,7^ Our lack of understanding of MSC immunobiology underlies the controversial results reported in multiple clinical trials showing unpredictable therapeutic efficacy of MSC infusions even in homogenously treated patients suffering from the same disease.^6,8,9^ Regardless of the MSC-specific phenotype and tissue of origin, the cell dose and administration schedule, the cell delivery route, and the selected cell product (ie, use of autologous vs allogeneic, freshly cultured vs frozen and thawed MSCs, etc.), a major unresolved challenge in the field is posed by the fact that MSCs do not seem required to engraft to be efficacious, as indicated by the fact MSCs are undetectable after in vivo administration.^10^ In fact, an increasing evidence in recent years indicates that extracellular vesicles (EVs) released by MSCs (MSC-EVs) might exert similar immunosuppressive effects as the MSC themselves.^11,12^ Such potential administration of MSC-EVs as a cell-free therapeutic approach may offer several therapeutic and safety advantages over the parental MSCs.^13^

A recent seminal study by Dazzi’s Lab based on preclinical murine models of GvHD as well as clinical data from MSC-infused GvHD patients demonstrated that the infused MSCs are actively induced to undergo apoptosis by recipient cytotoxic T cells, a mechanism that triggers in vivo recipient-induced immunomodulation by such apoptotic MSCs.^4,14^ In fact, this study also showed that the need for recipient cytotoxic cell activity could be replaced by the administration of apoptotic MSC generated ex vivo,^14^ further supporting the use of MSC-EVs as a cell-free therapeutic alternative over the parental MSCs.

The introduction of a “suicide gene” switch into MSCs may offer on-demand in vivo apoptosis of transplanted MSCs. This would potentiate the safety of the infused MSCs in the case of an adverse event but, will also circumvent current logistic technical limitations and biological uncertainties associated to MSC-EVs.^13^ The fusion “inducible Caspase-9” suicide gene (iCasp9) was recently engineered by replacing the Caspase recruitment domain of pro-apoptotic caspase-9 with a mutated dimerizer drug-binding domain from the human FK506-binding protein. The iCasp9 displays high affinity and high specificity for small molecular chemical inducers of dimerization (CID) such as clinical-grade AP1903, which effectively induces apoptosis via activation of caspase-9 without off-target unwanted effects.^15,16^ The iCasp-9 system comprises unmodified human components, and as shown in preclinical or clinical studies is unlikely to be immunogenic. Here, we prompted to investigate the utility of the iCasp9/AP1903 suicide gene system in inducing apoptosis of MSCs. iCasp9/AP1903-induced apoptotic MSCs (MSC^iCasp9+^) were tested in vitro and in in vivo models of acute colitis, and they show a robust immunosuppressive and anti-inflammatory properties in vitro and in vivo regardless of whether apoptosis was induced in vivo or in vitro before administering MSC^iCasp9+^ lysates. We report the successful development of an efficient iCasp9 switch for improved MSC-based therapies.

## Material and Methods

### Human Primary Cells

MSCs were sourced from both human BM aspirates from pediatric healthy donors obtained from the Catalan Blood and Tissue Bank (BST) and from cord blood-derived WJ upon Institutional Review Board approval (HCB/2014/0687). Both BM-MSCs and WJ-MSCs were generated and maintained using well-established procedures, and characterized morphologically, phenotypically, and functionally following the guidelines proposed by the International Society for Cellular Therapy.^17-19^ MSCs were cultured in complete Advanced DMEM medium (advanced DMEM supplemented with 10% heat-inactivated FBS, L-glutamine, and penicillin/streptomycin (P/S). *Bona fide* WJ-MSCs and BM-MSCs were used at early passages 1-6.

Peripheral blood mononuclear cells (PBMCs) were isolated from buffy coats of healthy volunteers by Ficoll-Hypaque gradient centrifugation (GE Healthcare, Chicago, IL) obtained from the BST upon Institutional Review Board approval (HCB/2014/0687). PBMCs were maintained in complete RPMI medium (RPMI-1640 supplemented with 10% heat-inactivated FBS and P/S (all from Gibco/Invitrogen, Waltham, MA).

### Vector Construction and Lentiviral Transduction of MSCs

**Figure 1.**
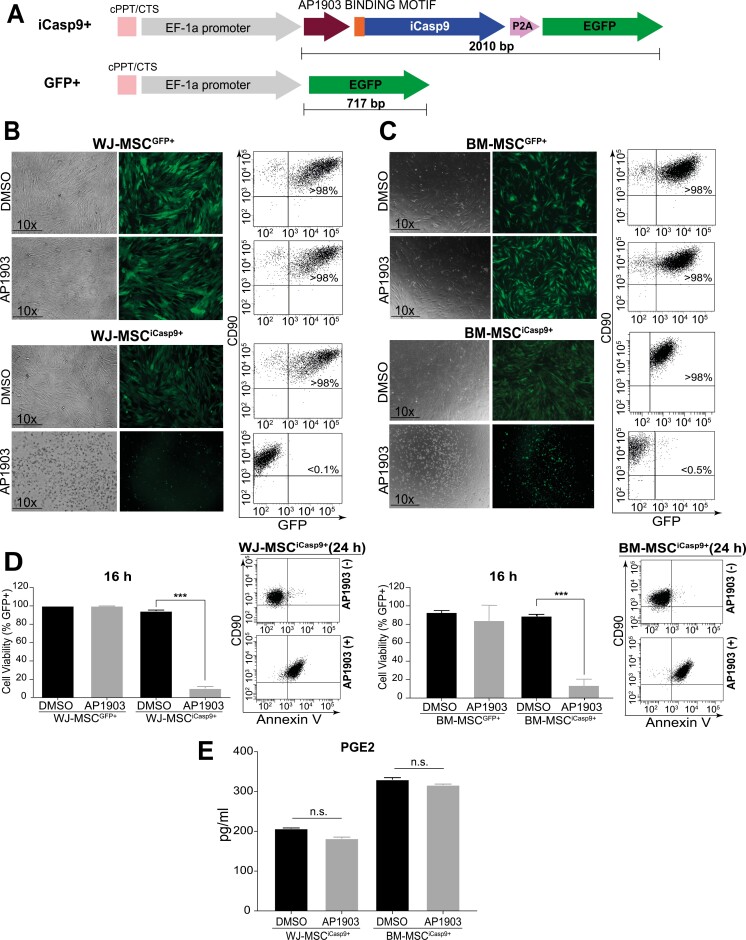
Rapid AP1903-mediated apoptosis of iCasp9-expressing WJ- and BM-MSCs. (**A**) Schematic of iCasp9/GFP-expressing lentiviral vector. A GFP-expressing vector was used as mock. The iCasp9 cDNA is preceded by an AP1903 binding motif for iCasp9 activation. (**B,C**) Cell viability measured by GFP expression assessed by fluorescence microscopy and flow cytometry in GFP-expressing (top panels) and iCasp9/GFP-expressing (bottom panels) WJ-MSCs (left) and BM-MSC (right) 16 hours and 24 hours after treatment with 20nM AP1903. Massive cell death (>95%) was exclusively observed in MSC^iCasp9+^ treated with AP1903 for 24 hours. (**D**) Quantification of cell death by GFP assessment after 16 hours and Annexin V staining after 24 hours, *n* = 5 independent experiments. Data shown as mean ± SEM. ∗∗∗*P* < .001 relative to DMSO. (**E**) ELISA quantification of PGE2 levels in WJ-MSC^iCasp9+^ and BM-MSC^iCasp9+^ supernatants harvested 24 hours after AP1903 treatment (*n* = 3). Data shown as mean ± SEM.

The AP1903 binding domain fused to iCasp9 (kindly provided by Prof Gianpietro Dotti, University of North Carolina, NC) before a 2A ribosomal skip sequence followed by a GFP reporter was subcloned downstream the EF1a promoter in our clinically validated pCCL second-generation lentiviral backbone^20,21^ (Fig. 1A). Similarly, the dTomato (dTo)-P2A-Luciferase (Luc) sequence was also subcloned downstream the UbC promoter in the pCCL vector (Supplementary Fig. S1A). iCasp9/GFP- or dTo/Luc-expressing lentiviral particles pseudotyped with VSV-G were generated by transfection of HEK 293T cells with pCCL, VSV-G, and psPAX2 vectors using standard polyethylenimine (Polysciences, Warrington, PA). Supernatants were collected 48 hours after transfection and concentrated by ultracentrifugation, as previously described.^22^

Semi-confluent WJ-MSCs and BM-MSCs were transduced in 6-well plates with iCasp9/GFP-expressing lentiviral particles (or GFP alone as a control) at a multiplicity-of-infection of 5 in the presence of polybrene (8-10 μg/mL).^23,24^ Viral supernatants were washed out 24 hours later, and transduced cells were allowed to recover and expand for 7-15 days. Then, the GFP+ (MSC^GFP+^) and iCasp9/GFP+ (MSC^iCasp9+^) transduced cells were FACS-purified (98% purity) using FACSAria Fusion flow cytometer (BD Biosciences, San Jose, CA). The WJ-MSC^iCasp9+^ cells were further transduced with Luc/dTo-expressing lentiviral particles, and the resulting WJ-MSC^iCasp9+Luc+^ were FACS-sorted at purity >94% and established as described above.^23,24^

### In Vitro AP1903 Apoptotic Assay in WJ and BM-MSC

MSC^GFP+^ and MSC^iCasp9+^ cells were plated in 6-well plates at 0.5×10^6^/well, and 48 hours later were treated with the CID AP1903 (20nM, Bellicum Pharmaceuticals, Houston, TX) and/or DMSO as the vehicle. Cells were analyzed by fluorescence microscopy and by flow cytometry 24 hours later, and the cell viability was assessed by Annexin V/7AAD apoptosis detection kit (BD Biosciences). Cell supernatants were harvested in triplicate and analyzed by ELISA assay for quantification of prostaglandin E2 (PGE2).^24^

### In Vitro Immunosuppression of T-cell Response by MSC^GFP+^ and MSC^iCasp9+^ and Cytokine Production

MSC^GFP+^ and MSC^iCasp9+^ cells (both WJ and BM) were seeded at 2×10^4^ cells/well in flat-bottom 96-well plates containing complete Advanced DMEM medium for 24 hours. The next day, 1×10^6^ cells PBMCs were labeled with 2.5 μM CFSE for 20 minutes at 37°C and then washed 3× with complete RPMI medium. Subsequently, the MSC culture medium was removed and CFSE-labeled PBMCs were added to the MSC culture at MSC:PBMC ratios of 1:5 and 1:10 in complete RPMI medium in the absence or presence of PHA-L (1 μg/mL). The day after, AP1903 (20nM) or DMSO as vehicle was added, and 96 hours later the non-adherent cells were collected, washed and stained with 7-AAD. Cell proliferation was measured using CSFE dye dilution on the 7-AAD- viable cells by FACS and the number of proliferating cells was determined by gating on the CFSE^low^ subset as previously described.^25,26^ PBMC:MSC co-cultures supernatants were harvested and analyzed in triplicate by ELISA assay for quantification of the pro-inflammatory cytokines IL-2 and IL17A.

### Induction, Treatment, and Analysis of Inflammatory Bowel Disease

The animal experiments reported in this study were approved by the Animal Care and Ethical Committee of Institute of Parasitology and Biomedicine “López-Neyra”-Spanish Council of Scientific Research (IPBLN-CSIC, code number: MDM.03.10/17.CEEA) and performed in compliance with the guidelines from Directive 2010/63/EU of the European Parliament on the protection of animals used for scientific purposes. Balb/c mice were purchased from Charles River and were housed in the specific pathogen-free animal facility of IPBLN-CSIC in a controlled-temperature/humidity environment (22±1°C, 60%-70% relative humidity) in individual cages (10 mice per cage, with wood shaving bedding and nesting material), with a 12-hour light/dark cycle and were fed with rodent chow (Global Diet 2018, Harlan) and tap water ad libitum.

To induce acute colitis, 2,4,6-trinitrobenzene sulfonic acid (TNBS, 2.5mg) in 50% ethanol (100 μL) was administered intrarectally in 6-8-week-old female Balb/c mice (125mg of TNBS/kg mouse) under light halothane-induced anesthesia.^27,28^ Control mice received 50% ethanol alone. Animals were treated intra-peritoneally (i.p.) with DMEM medium (untreated mice) or with WJ-MSC^GFP+^ or WJ-MSC^iCasp9+^ (10^6^ cells/mouse, in a volume of 200 μL of DMEM) 12 hours after instillation of TNBS. Eight hours later, WJ-MSC^iCasp9+^-treated animals were injected i.p. with vehicle (1% DMSO in 100 μL of DMEM) or AP1903 (5mg/kg, in 100 μL of DMEM). Additionally, in another group of animals, treatment consisted of the injection i.p. of cell lysates (in a volume of 200 μL of DMEM) obtained from overnight treatment with AP1903 (20nM) of WJ-MSC^iCasp9+^ cultures (10^6^ cells).

Animals were daily monitored for the appearance of diarrhea, body weight loss, and survival. At different time points, colitis signs were scored based on stool consistency and rectal bleeding by 2 blind observers using the following criteria (scale 0-4): 0 = normal stool appearance; 1 = slight decrease in stool consistency; 2 = moderate decrease in stool consistency; 3 = moderate decrease in stool consistency and presence of blood in stool; and 4 = severe watery diarrhea and moderate/severe bleeding in the stool.^25,28,29^ At days 4 and 9 after TNBS injection, sera were collected by cardiac puncture, and colons were dissected from caecum to the anus. The levels of the inflammatory cytokines TNFα and IL6 in the sera were determined by specific ELISAs as previously described.^25,27-29^ Colons were macroscopically scored for tissue damage signs (scale 0-8), based on the grade of tissue adhesion, presence of ulceration and wall thickness: ulceration (0, normal appearance; 1 = focal hyperemia, no ulcers; 2 = ulceration without hyperemia or bowel wall thickening; 3 = ulceration with inflammation at one site; 4 = two or more sites of ulceration and inflammation; and 5 = major sites of damage extending >1cm along length of colon), adhesions (0 = no adhesions; 1 = minor adhesions, colon can be easily separated from the other tissues; and 2 = major adhesions) and thickness (maximal bowel wall thickness, in mm, measured with a caliper).^25,27-29^ After macroscopic examination, various colonic segments were fixed in 10% buffered formalin phosphate, paraffin-sectioned (5 µm), stained with Masson’s trichrome and Russell-Movat pentachrome solutions and histopathologically analyzed in an Axio Scope A1 microscope (Carl Zeiss) in a blind fashion by 2 independent researchers (5 cross-sections per animal, at 2-mm intervals along colon segment, at 100× magnification). Intestinal inflammation in colon sections was graded from 0 to 4 as follows: 0 = no signs of inflammation; 1 = low leucocyte infiltration (1-2 foci); 2 = moderate leucocyte infiltration with multiple foci; 3 = high leucocyte infiltration, moderate fibrosis, high vascular density, thickening of the colon wall, moderate goblet cell loss and focal loss of crypts; and 4 = transmural infiltrations, massive loss of goblet cells, extensive fibrosis and diffuse loss of crypts.^25,27,29^ Alternatively, inflammatory infiltration was quantified in a blinded fashion by counting the number of infiltrating cells in colon mucosa in 5 independent cross-sections using 8 randomly selected nonoverlapping high-powered fields (hpf, at 400× magnification) per section and expressed as the mean of leucocytes/hpf.

### Statistical Analysis

All values are expressed as mean ± SEM of mice/experiment. For statistical analysis, Prism GraphPad Prism v9.0 (GraphPad Prism Software, San Diego, CA) was used. The differences between groups (single comparisons) were analyzed by the nonparametric Mann-Whitney *U* test (for analysis of colon damage, colitis, and microscopic scores) or by the unpaired Student’s *t* test (for analysis of cell viability, PBMC proliferation, cytokine levels, and changes in body weight). One-way analysis of variance with Tukey’s post hoc test was used when 3 or more experimental groups were compared. Survival curves were analyzed by the Kaplan-Meier log-rank test. We considered *P*-values <.05 (2-tailed) as significant. No data were excluded from the analysis. The variance was similar between the groups that were statistically compared.

## Results

### Treatment with the CID AP1903 Induces Rapid and Efficient Apoptosis of WJ-MSC^iCasp9+^ and BM-MSC^iCasp9+^

Early passage, fully characterized WJ- and BM-MSCs^17,19^ were transduced with iCasp9/GFP-expressing (or GFP alone as control) lentivectors (Fig. 1A). Transduced MSCs were FACS-sorted based on GFP expression at high purity (>95%, Fig. 1B,C). When exponentially growing MSC^GFP+^ and MSC^iCasp9+^ were treated with AP1903 (20nM), a massive (approximately 85%-100%) apoptosis/cell death was observed within 16-24 hours in both WJ- and BM-MSC^iCasp9+^ but not in WJ- and BM-MSC^GFP+^ (Fig. 1B-D). The levels of PGE2, a major *bona fide* immunosuppressive factor produced by MSCs, were very similar in the supernatants of both alive (DMSO-treated) and apoptotic (AP1903-treated) WJ- and BM-MSC^iCasp9+^ (Fig. 1E), confirming a sensitive and specific performance of the iCasp9/AP1903 switch in human MSCs, in line with that previously reported for induced pluripotent stem cells,^16^ T-cells,^30^ and MSC-based cancer gene therapies.^31,32^

### Apoptotic WJ- and BM-MSC^iCasp9+^ Robustly Immunosuppress T-cell Response In Vitro

**Figure 2.**
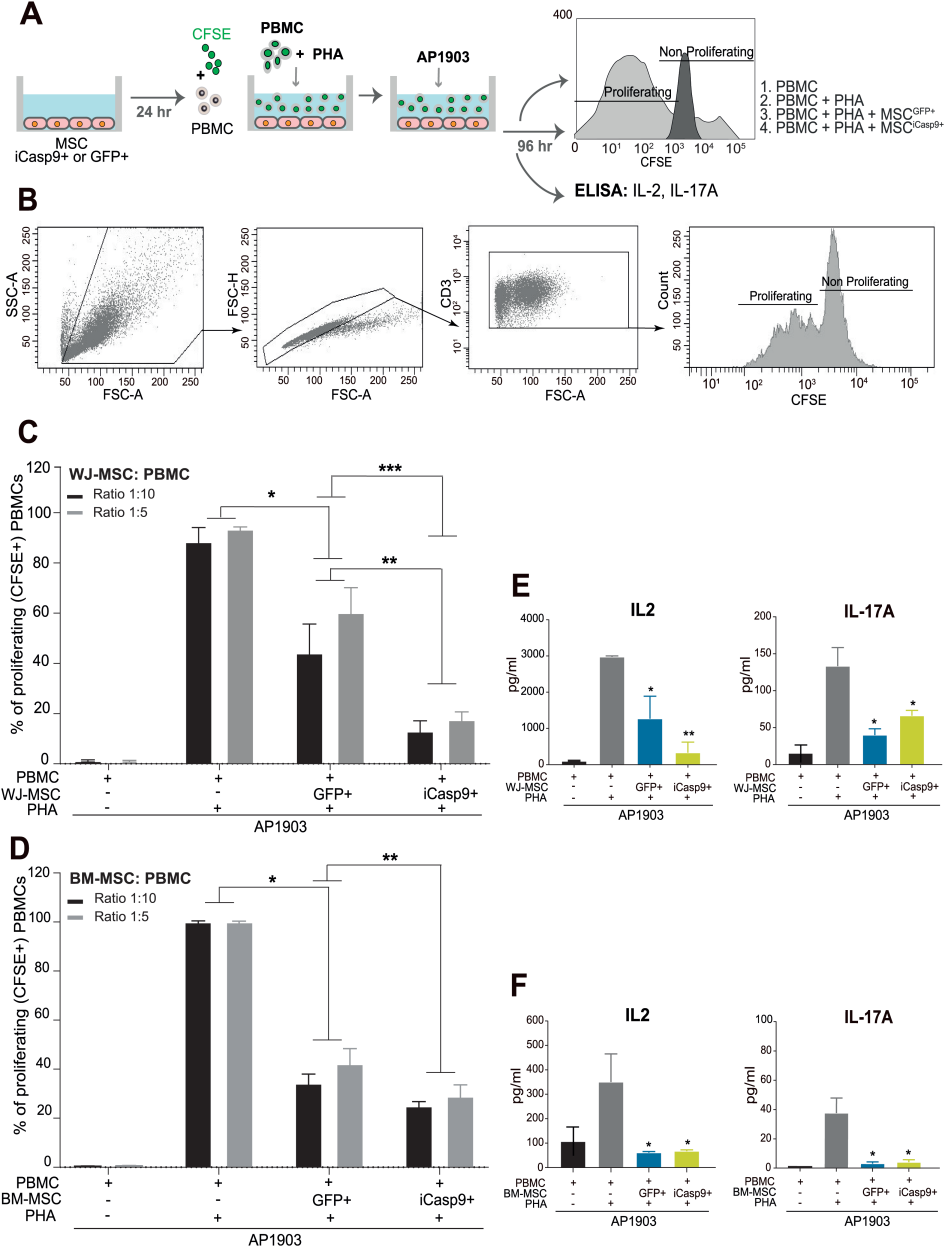
iCasp9/AP1903-induced apoptotic WJ- and BM-MSC robustly immunosuppress T-cell response. **(A**) Scheme of in vitro T-cell immunomodulation assays with AP1903-treated MSC^iCasp9+^ and MSC^GFP+^. (**B**) Boolean gating flow cytometry strategy to identify proliferating T-cells. (**C**) Percentage of proliferating T cells measured as % of CFSE+ T cells 6 days after PHA-L stimulation in the absence or presence of AP1903-treated WJ-MSC^iCasp9+^ and WJ-MSC^GFP+^. 1:5 and 1:10 MSC:PBMC ratios were used. (**D**) Identical to panel B but for BM-MSC^iCasp9+^ and BM-MSC^GFP+^. (**E,F**) ELISA quantification of the pro-inflammatory cytokines IL-2 and IL-17A in WJ-MSC:PBMCs (E) and BM-MSC:PBMCs (F) supernatants after 6 days at 1:10 MSC:PBMC ratio. Data show as mean ± SEM. One-way ANOVA test with Turkey’s post hoc test. ∗*P* < .05, ∗∗*P* < .01, ∗∗∗*P* < .001.

MSC are widely recognized for their immunomodulatory potential, including the inhibition of allogenic T-cell proliferation and the production of pro-inflammatory cytokines.^33,34^ We therefore monitored PHA-L-stimulated T-cell division in the absence or presence of AP1903-treated WJ- and BM-derived MSC^GFP+^ and MSC^iCasp9+^ in vitro (Fig. 2A,B). We found that apoptotic (AP1903-treated) WJ- and BM-MSC ^iCasp9+^ strongly inhibited T-cell proliferation, even at a significantly higher extent than alive WJ- and BM-MSC^GFP+^ (Fig. 2C,D). We next analyzed MSC:T-cell supernatants to test whether apoptotic MSC^iCasp9+^ also regulates pro-inflammatory cytokine secretion. The analysis of supernatants showed that the levels of IL-2 and IL-17A were massively reduced in the presence of apoptotic WJ- and BM-MSC^iCasp9+^ (Fig. 2E,F). Overall, these results show that regardless of the source of origin, the capacity of apoptotic MSC^iCasp9+^ to immunosuppress T-cell response in vitro is as robust as that displayed by alive MSC^GFP+^ cells.

### Robust In Vivo Anti-inflammatory Properties of Apoptotic MSC^iCasp9+^ and MSC^iCasp9+^ Lysates in a Mouse Model of Acute Colitis

**Figure 3.**
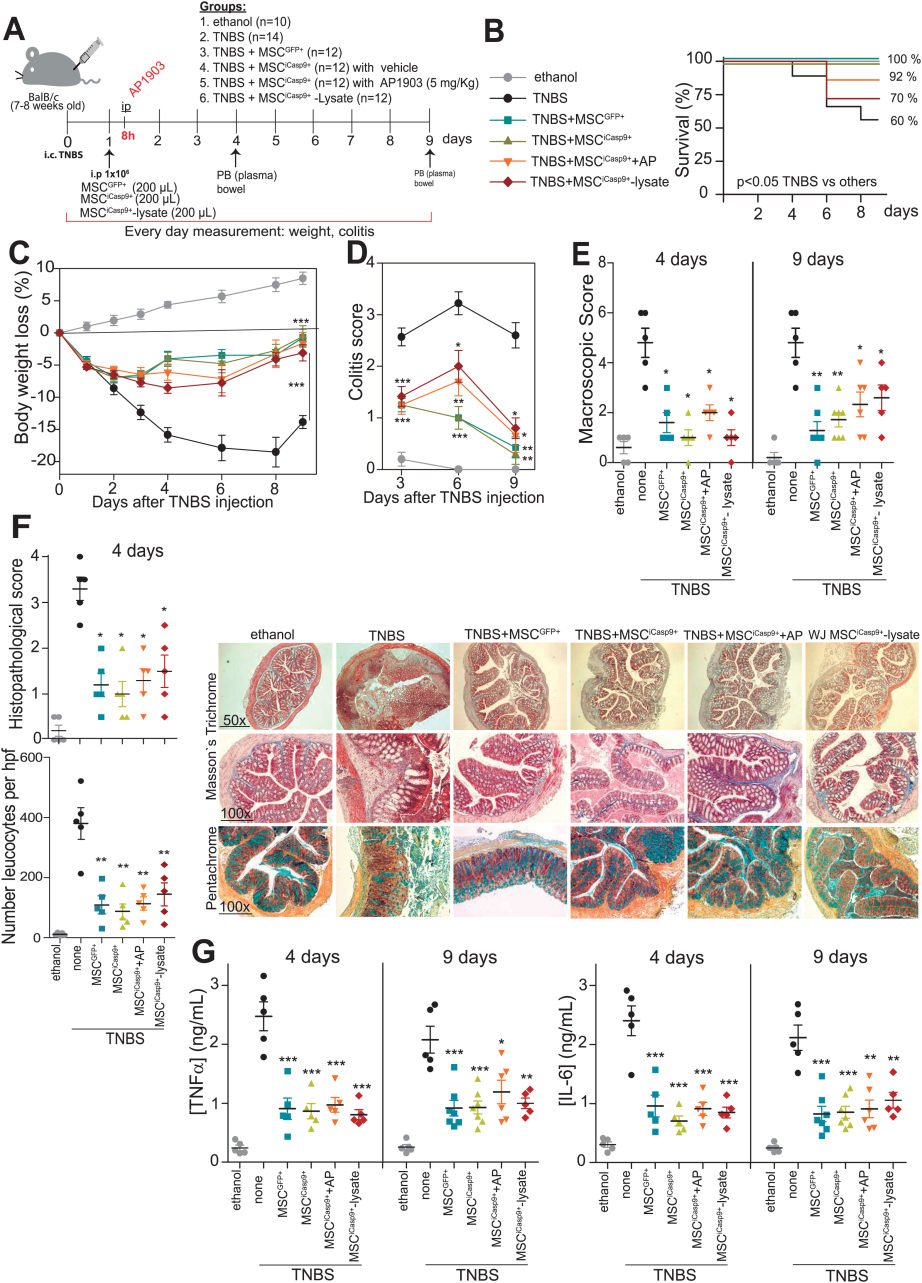
In vivo anti-inflammatory properties of iCasp9/AP1903-induced apoptotic MSCs and MSC^iCasp9+^ lysates in a mouse model of acute colitis. (**A**) Scheme of the experimental design. Acute colitis was induced in 7-8-week-old mice BalB/c by intracolonic administration of TNBS or ethanol (control). After 24 hours, mice (*n* = 10-14 per group) were i.p. injected with medium or MSC^GFP+^ or MSC^iCasp9+^ (10^6^ cells/mouse). Eight hours after i.p. infusion of MSC^iCasp9+^ cells, mice were i.p. injected with either vehicle or AP1903 (5mg/kg). Body weight and survival were followed up for 9 days. WJ-MSC^iCasp9+^ cells (10^6^) were also treated in vitro with 20nM AP1903 for 24 hours, and then, the WJ-MSC^iCasp9+^ lysate was i.p. infused in TNBS-treated mice. **(B**) Kaplan-Meyer curves showing a significantly improved survival in mice treated with MSC^GFP+^ or MSC^iCasp9+^ +/-AP1903, but not with MSC^iCasp9+^ lysates, compared to TNBS alone group. (**C**) Daily change in body weight loss relative to day 0 (induction of colon damage). (**D**) Colitis score determined at days 3, 6, and 9. (**E**) Macroscopic damage score of the intestine evaluated at days 4 and 9 for the indicated treatments. (**F**) Left, histopathological scores of colons and number of infiltrating leucocytes in colonic mucosa determined at day 4 for the indicated treatments. *Right,* representative colon sections stained with Masson’s trichrome and Russell-Movat pentachrome for the indicated treatments. (**G**) Serum levels of TNFα and IL-6 at days 4 and 9 for the indicated treatments. Data are shown as mean ± SEM. ∗*P* < .05, ∗∗*P* < .01, ∗∗∗*P* < .001 compared to TNBS, 1-way ANOVA test with Turkey’s post hoc test.

Having confirmed the immunosuppressive properties of apoptotic MSC^iCasp9+^ in vitro, we next assessed their anti-inflammatory properties in vivo, by harnessing a well-established preclinical model of acute colitis that shares clinical, histopathological, and immunological features with Crohn’s disease (Fig. 3A).^35,36^ To facilitate the in vivo tracing, WJ-MSC^iCasp9+^ cells were transduced with dTo/Luc-expressing lentiviral particles, and the GFP+/dTo+ double-positive MSCs (WJ-MSC^iCasp9+Luc+^) were FACS sorted at high purity (Supplementary Fig. S1A-C). Inflammatory colitis was induced by intracolonic administration of TNBS, and mice were i.p. infused with either DMEM medium (untreated mice) or with 10^6^ WJ-MSC^GFP+^ or WJ-MSC^iCasp9+/Luc+^ 12 hours after TNBS instillation. WJ-MSC^iCasp9+/Luc+^-treated animals were *i.p* treated 8h later with either vehicle or AP1903. In line with the in vitro results, in vivo administration of AP1903 successfully induced rapid ablation/apoptosis of WJ-MSC^iCasp9+/Luc+^ (Supplementary Fig. S1D,E). As expected, TNBS-treated mice developed severe, an acute illness characterized by 40% mortality over a 9-day period (Fig. 3B) accompanied by substantial (approximately 20%) and sustained body weight loss (Fig. 3C), bloody diarrhea, rectal prolapsed, and pancolitis with the extensive wasting syndrome (Fig. 3D). Macroscopic examination of colons revealed profound signs of inflammation, hyperemia, ulceration, and shortening (Fig. 3E). In contrast, mice treated with apoptotic WJ-MSC^iCasp9+/Luc+^ (infusion of WJ-MSC^iCasp9+/Luc+^ followed by i.p. treatment with AP1903) were largely protected against colitis, similar to those mice treated with alive MSC^GFP+^ cells, showing a significant recovery of their body weight loss, with a significant increase in survival rate (60% vs 92%) that was accompanied by the regaining of a healthy appearance, indistinguishable from ethanol-treated control mice (Fig. 3B,C). Furthermore, the wasting syndrome and the signs of colon inflammation were equally improved in mice treated with either apoptotic WJ-MSC^iCasp9+/Luc+^ or MSC^GFP+^ cells (Fig. 3D,E). Colitis and colon macroscopic score data were directly supported by histological colonic examination which showed that apoptotic WJ-MSC^iCasp9+/Luc+^ significantly reduced TNBS-induced transmural inflammation, depletion of mucin-producing goblet cells, epithelial ulceration, infiltration of inflammatory cells in the lamina propria, and focal loss of crypts (Fig. 3F). To further confirm the in vivo anti-inflammatory nature of apoptotic WJ-MSC^iCasp9+/Luc+^, we measured at day 4 and day 9 after TNBS instillation the levels of the pro-inflammatory cytokines TNFα and IL6 in the serum and found a pronounced decrease in the levels of both cytokines in colitis mice treated with apoptotic WJ-MSC^iCasp9+/Luc+^ as compared to untreated mice (Fig. 3G). Importantly, no statistically differences were observed among mice treated with apoptotic WJ-MSC^iCasp9+/Luc+^ and those treated with alive WJ-MSC^iCasp9+/Luc+^ (without in vivo treatment with AP1903) or WJ-MSC^GFP+^ (Fig. 3). Finally, very similar therapeutic effects were observed in colitis mice *i.p* treated with WJ-MSC^iCasp9+/Luc+^ cell lysates obtained from overnight treatment of WJ-MSC^iCasp9+/Luc+^ cells with AP1903 (Fig. 3). Collectively, our results show that apoptotic WJ-MSC^iCasp9+/Luc+^ are equally capable of suppressing inflammation in vivo than MSC^GFP+^ cells, largely protecting mice against acute colitis in a TNBS-induced disease model.

## Discussion

MSC therapies are clinically used in a wide range of disorders. However, although the therapeutic efficacy of MSCs has been shown in preclinical animal studies and in human phase I/II clinical trials, progress beyond phase III trials has not been achieved.^6^ A major challenge in the clinical implementation of MSC therapies is posed by the lack of understanding about the mechanisms underlying MSC therapeutic activity as demonstrated by the unpredictable therapeutic efficacy of MSC infusions reported in multiple clinical trials. In addition, a major unresolved challenge in the field is posed by the fact that MSCs do not seem required to engraft to be efficacious, as shown by undetectable MSCs after in vivo infusion.^10^ Increasing evidence also suggests the use of MSC-EVs as a cell-free therapeutic alternative with several advantages over alive, parental MSCs. However, many biological and scale-up challenges still need to be outdone. In particular, the efficacy and amenability of MSC-EVs are far from being (pre)-clinically confirmed.^13,37^ Moreover, preclinical data suggests an inferior biological effect of MSC-EVs over alive “parental” MSCs likely because MSCs exert their function not only through EVs but also by direct cell-to-cell contact and through paracrine factors released by MSCs beyond those exchanged in EVs.^38^

Previous seminal work from Dazzi’s Lab demonstrated that apoptotic MSCs induce in vivo recipient-mediated immunomodulation and that among MSC-treated patients only those with high cytotoxicity activity against infused MSCs responded well to MSC therapy, thus representing a paradigm shift in MSC-based therapies.^4^ Furthermore, the need for such recipient cytotoxic cell activity could be replaced by the administration of ex vivo-generated apoptotic MSCs.^4^ In the present study, we have developed an iCasp9-based safety switch for safer MSC-based therapies. iCasp9/AP1903-induced apoptotic MSCs (MSC^iCasp9+^) were tested in vitro and in in vivo in cutting-edge models of acute colitis. Our data show a robust immunosuppressive and anti-inflammatory properties of MSC^iCasp9+^ in vitro and in vivo regardless of whether apoptosis was induced in vivo or in vitro before administering MSC^iCasp9+^ lysates. Apoptotic MSCs are expected to be engulfed by recipient phagocytes, without affecting the immunosuppressive therapeutic potential of the MSC cargo.^4^ Although there are more suicide genes, the iCas9 system has obvious advantages namely: (1) the iCasp9 displays high affinity and high specificity for small molecular CIDs which effectively induce apoptosis via activation of caspase-9 without off-target unwanted effects,^16,31^ (2) the iCasp-9 system comprises unmodified human components, and as shown in preclinical or clinical studies is unlikely to be immunogenic. However, all other suicide gene systems contain yeast, bacterial or viral expressed proteins, and are likely to be immune targets and noncompatible with long-term persistence in target organs/tissues. The efficiency and specificity of the iCasp9/AP1903 switch here reported in human MSCs is in line with that previously reported for induced pluripotent stem cells,^16^ T-cells,^30^ and MSC-based cancer gene therapies.^31,32^ This approach will become of increasing value as clinical applications for MSCs develop further.

## Conclusion

Despite the similar efficacy of both “parental” alive MSC^GFP+^ cells and apoptotic MSC^iCasp9+^ cells, this development of an efficient iCasp9 switch may potentiate the safety of MSC-based therapies in the case of an adverse event, offering and strategy to circumvent current logistic and technical limitations and biological uncertainties associated to both alive “parental” MSCs and MSC-EVs. The present development of an efficient iCasp9 switch strategy may pave the way to new avenues in the clinical manufacturing of MSCs. In particular, apoptotic MSCs should not be subject to FDA or EMA regulations for advanced cell therapies since they may well be classified a cell-free therapy and considered under the pharmaceutical class of biologics.^13^ Furthermore, like MSC-EVs, apoptotic MSCs will be safe as they will not have the risk of ex vivo or in vivo oncogenic transformation themselves.^24,39-41^

## Supplementary Material

szab007_suppl_Supplementary_Figure_S1

## Data Availability

The data underlying this article are available in the article and in its online supplementary material.
