# Supplementary figures and images for: Robust In Vitro and In Vivo Immunosuppressive and Anti-inflammatory Properties of Inducible Caspase-9-mediated Apoptotic Mesenchymal Stromal/Stem Cell
Source: Stem Cells Transl Med. 2022 Mar 3;11(1):88–96. doi: 10.1093/stcltm/szab007 (PMC8895490; doi:10.1093/stcltm/szab007)

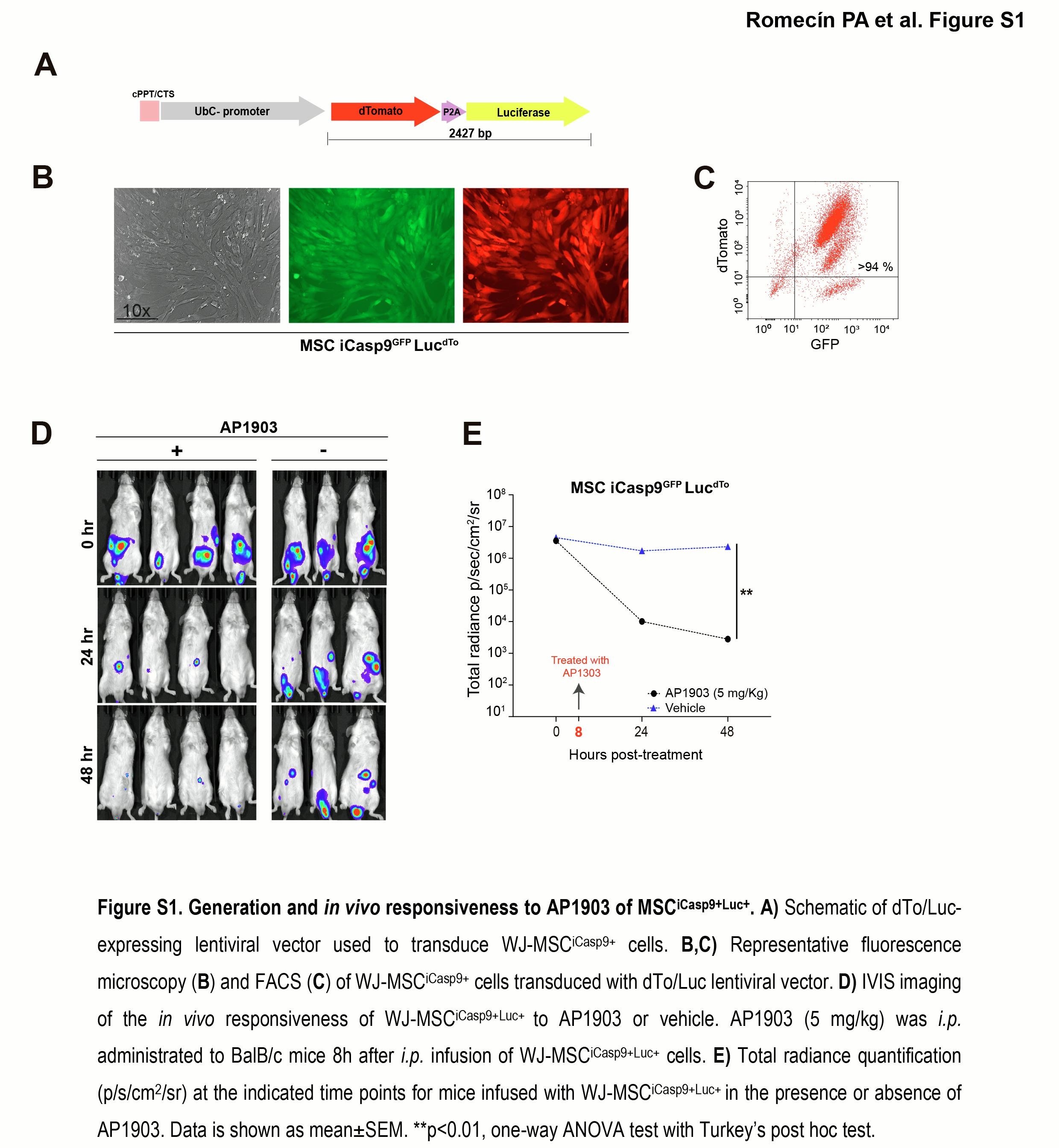

Supplement: szab007_suppl_Supplementary_Figure_S1 [file szab007_suppl_supplementary_figure_s1.jpeg]
